# RSS Fingerprint Based Indoor Localization Using Sparse Representation with Spatio-Temporal Constraint

**DOI:** 10.3390/s16111845

**Published:** 2016-11-03

**Authors:** Xinglin Piao, Yong Zhang, Tingshu Li, Yongli Hu, Hao Liu, Ke Zhang, Yun Ge

**Affiliations:** 1 Beijing Advanced Innovation Center for Future Internet Technology, Beijing Key Laboratory of Multimedia and Intelligent Software Technology, Beijing University of Technology, Beijing 100124, China; piaoxinglinphd@emails.bjut.edu.cn (X.P.); zhangyong2010@bjut.edu.cn (Y.Z.); yuqingqing@emails.bjut.edu.cn (T.L.); geyun@bjut.edu.cn (Y.G.); 2 Beijing Transportation Information Center, Beijing 100073, China; hao.liu@bjjtw.gov.cn; 3 Beijing Transportation Coordination Center, Beijing 100073, China; zhangke@bjjtw.gov.cn

**Keywords:** indoor localization, RSS fingerprint, sparse representation, temporal constraint, spatial constraint

## Abstract

The Received Signal Strength (RSS) fingerprint-based indoor localization is an important research topic in wireless network communications. Most current RSS fingerprint-based indoor localization methods do not explore and utilize the spatial or temporal correlation existing in fingerprint data and measurement data, which is helpful for improving localization accuracy. In this paper, we propose an RSS fingerprint-based indoor localization method by integrating the spatio-temporal constraints into the sparse representation model. The proposed model utilizes the inherent spatial correlation of fingerprint data in the fingerprint matching and uses the temporal continuity of the RSS measurement data in the localization phase. Experiments on the simulated data and the localization tests in the real scenes show that the proposed method improves the localization accuracy and stability effectively compared with state-of-the-art indoor localization methods.

## 1. Introduction

In recent years, with the growing applications of Location-Based Service (LBS), wireless localization technology, especially the indoor wireless localization technology becomes an important research topic in wireless network communications. The main goal of indoor localization is to make the mobile terminal (e.g., a smart phone) obtain the location of itself and provide position information for users. Current indoor localization methods can be roughly divided into three types: (1) localization methods based on special equipment [1,2], which measure the location by using special equipment, such as active bats; (2) the wireless signal ranging methods [3], which measure the location by range measurements such as the Time Of Arrival (TOA) localization method; (3) the methods based on Signal Strength Fingerprint Maps (SSFM) [4,5,6], which first collect the wireless signal strengths of the scene and construct the scene fingerprint maps and then match the observed signal intensity of the mobile terminal with the fingerprint maps to obtain the location. Compared with the first and second indoor localization methods, the fingerprint-based methods fully utilize the existing wireless network resource, which is a common infrastructure in many places, and receive the signal strength from the MAC layer without any additional sensors on the mobile terminal. Moreover, due to the utilization of the inherent fingerprint data, which depend on the feature of the place, the fingerprint-based methods usually provide high localization accuracy. Therefore, the fingerprint-based methods are considered as a prospective and dominant indoor localization methods.

Generally, the localization procedure of the fingerprint-based methods can be divided into two stages, namely the off-line fingerprint maps construction stage and the online localization stage. In the off-line fingerprint maps construction stage, we collect the Received Signal Strength (RSS) at different positions in the given place and record the corresponding coordinates of the positions simultaneously. These data are then represented as a database called fingerprint maps of the place. In the online localization stage, we measure the RSS on the walking path by a mobile terminal and match the measurement with the fingerprint maps to find out the approximated signal strengths. By the coordinates of these approximated RSS strengths, the location of the measurement can be estimated. The key problem of the above localization is how to effectively find the matched or approximated RSS strengths in the fingerprint maps and estimate the position of the measurement with high precision. To overcome this problem, many researchers have proposed various localization algorithms, such as the KNN method [7,8,9], the Sparse Representation (SR)-based method [10], the Compressed Sensing (CS)-based method [11,12,13,14,15], etc. Although these fingerprint-based localization methods obtain acceptable positioning performance, most of the current localization methods do not explore and utilize the spatial correlation properties among fingerprint maps, as well as the temporal continuity of the measurements when the user is moving in his/her path. This results in many disadvantages of the current fingerprint-based localization methods, such as lacking robustness to noises and outliers and having limited localization accuracy. Therefore, some temporally- or spatially-constrained RSS localization methods were proposed. Ferris et al. [16] proposed a technique for solving WiFi-SLAM, which uses the Gaussian process latent variable models to relate RSS fingerprints and models human movements (displacement, direction, etc.) as hidden variables. Huang et al. [17] proposed a method named GraphSLAM, which further improves WiFi-SLAM regarding computing efficiency and relying assumptions. In these two methods [16,17], the signal measurement likelihoods are modeled as Gaussian random variables, and so, the similarities in both the temporal and spatial domain are utilized in the localization.

In this paper, we propose an RSS fingerprint-based indoor localization method by using a revised sparse representation model, namely the Spatio-Temporal Sparse Representation model (ST-SR), which integrates the spatio-temporal correlation in RSS fingerprint maps and the RSS measurements in the localization procedure. In the proposed method, the inherent spatial correlation of fingerprint maps and the temporal continuity of the RSS measurement data are modeled as spatio-temporal constraints, which is similar to [16,17], and integrated into a traditional sparse representation model. The main contribution of the proposed method is that the ST-SR model gives a proper way to combine the traditional SR method with the spatial correlation among the RSS fingerprint data and the temporal continuity of testing RSS measurements, which reveal the intrinsic properties of the data involved in indoor localization. Additionally, to solve the complicated optimization problem with multiple constraints in the proposed model, we propose an effective algorithm as the solution. To evaluate the proposed method, several localization experiments are implemented on both the simulated data and the real scenes. The experimental results demonstrate that the proposed method achieves higher localization accuracy with good stability and robustness compared with state-of-the-art indoor localization methods.

The rest of the paper is organized as follows. In Section 2, we summarize the related works of wireless localization methods. Section 3 introduces the basic SR model-based localization method. Section 4 presents the proposed ST-SR model-based localization method. Section 5 gives the solution to the optimization problem of the proposed ST-SR model. We will show the experimental results of our proposed methods compared with state-of-the-art methods in both simulated and real scenes in Section 6. Section 7 concludes the paper with a discussion on future research.

## 2. Related Works

For the past few years, researchers have proposed many indoor localization methods, including the methods based on special equipments, the methods based on wireless signal ranging and the SSFM-based methods. The methods based on special equipment were proposed earliest among all indoor localization methods, which generally need special equipment, such as active bats. Want et al. [1] proposed the first localization system called the Active Badge System (ABS). In this system, infrared signals are periodically broadcast through the moving unique transmitters and transmitted to a central server for estimating position. Harter et al. [2] proposed the Bat Localization System (BLS) in which wireless and ultrasonic technology were adopted. BLS is comprised of bat nodes, ultrasonic receiving units and a central database. The receiving units have previously been placed at known positions and form an interconnected array through the wired network. The bat nodes periodically broadcast their own ID and transmit ultrasonic pulses. The receiving unit records the arrival time of the radio signal and the ultrasonic signals from nodes. The localization is implemented by calculating the distances according to the propagation speed of the radio waves and the sound waves in the air. There are some other localization methods, such as the Distributed Indoor Localization Systems Cricket (DILSC) [18], the VHF-round distance (VHF omnidirectional ranging) [19], the Ultra-Wide Band (UWB)-based method [20], the Radio Frequency Identification (RFID) tag-based method [21] and the ZigBee-based method [22]. Though these localization methods have achieved great success in localization applications and obtained high localization accuracy, they generally depend on dedicated or high-cost hardware facilities, which limits their applications in real scenes.

Recently, researchers have focused on the localization technology based on wireless signal strength and proposed some effective localization methods, such as the Angle Of Arrival (AOA)-based localization method [3], the Time Of Arrival (TOA)-based localization method [23], the Time Difference Of Arrival (TDOA)-based localization method [20] and the wireless signal propagation model-based method [24]. AOA relies on the measured angles relative to multiple base stations to find the position of the mobile device. However, it is sensitive to environmental factors in the localization processing, such as signal noise. TOA, the basis of the GPS system, calculates the distances between Light-Emitting Diodes (LEDs) and mobile devices from the arrival time of signals and then uses these estimated distances to derive the position of the mobile device. Although TOA could estimate the location with high accuracy, the method usually requires a direct path of signal propagation between LEDs and mobile devices. However, the direct path is difficult to guarantee in a real environment. In addition, it needs the corresponding hardware equipment to ensure the synchronization of signal propagation, which would have a high cost for localization. TDOA is an improved localization method of TOA. It determines the position of the mobile device based on the time difference of arrival of signals from multiple LEDs. However, it relies heavily on the distance of signal transmission. The above localization methods based on the wireless signal propagation model generally adopt the ideal wireless signal transmission model to describe the spatial variation of the signal intensity. Nevertheless, the wireless signal transmission is easily influenced by the complicated indoor environment. Therefore, when the testing scene changed, the localization methods based on wireless signal ranging could not obtain stable localization results.

The SSFM-based localization method is an attractive topic in wireless indoor localization [25,26,27,28,29]. Chang et al. [25] integrated Pedestrian Dead Reckoning (PDR) with WiFi fingerprinting to provide an accurate positioning algorithm. Park et al. [26] proposed a method to collect off-line data effectively in a fingerprinting-based indoor location estimation system based on using Kalman filtering. Wang et al. proposed some effective fingerprinting-based indoor location methods, such as the surface fitting technique-based indoor localization method [27] and the Curve Fitting (CF) and location search-based indoor localization scheme [28]. For reducing the computation complexity during the localization process, Wang et al. [29] proposed a new indoor subarea localization scheme via fingerprint crowdsourcing, clustering and matching, which first constructs subarea fingerprints from crowdsourced RSS measurements and relates them to indoor layouts. Since this localization method does not need any additional hardware, it is a low-cost and easily executed localization technology. Additionally, the localization method based on RSS fingerprint maps makes full use of the wireless signals changing within specific scenes, so it is considered as a context-adapting localization method and can be applied in different scenes. As described in the above section, having constructed the RSS fingerprint maps, the most important step of the SSFM-based localization method is the online localization, which is a problem of matching the observed RSS measurement to the database of RSS fingerprint maps. To solve this issue, many matching methods are proposed, including the KNN method [7,8,9], the Sparse Representation (SR)-based method [10] and the compressed sensing-based method [11,15]. The KNN method is a simple algorithm that selects the *K* closest RSS strengths from the fingerprint maps according to the similarity of the signal strength and computes the location by a specific weighted sum of the coordinates of the *K* RSS strengths. The SR-based method [10] is proposed based on sparse representation theory [30,31], in which the measurement is supposed to be sparsely represented by the RSS strengths in the fingerprint maps as it is only related to the RSS samples in its neighborhood. However, neither KNN nor SR considers the spatial distribution of RSS fingerprint data and the temporal continuity of the user’s measurements. The Compressed Sensing (CS) theory provides a new avenue for localization application. According to the CS theory, a sparse signal can be accurately reconstructed with a relatively small number of measurements [32]. From this principle, Feng et al. and Li et al. proposed a CS-based indoor localization method [11,15]. In this method, the RSS samples in the fingerprint maps are first clustered into several subsets according to their spatial correlation. Then, the distances between the observed RSS measurement and each cluster center are calculated, and the observed RSS measurement is classified into the most suitable subset of fingerprint maps with the minimum distance to the cluster center. Finally, the sparse representation of the RSS measurement is obtained by the CS algorithm in [11,12,13,14], and the final location is estimated by the positions of the RSS samples with non-zero sparse coefficients. The CS-based localization method utilizes the spatial correlation of the RSS fingerprint maps and produces better localization performance compared with KNN. Yet, this method ignored the temporal continuity of the observed RSS measurements.

## 3. Localization Method Based on Sparse Representation

In order to describe the localization method based on the proposed spatio-temporal constraint sparse representation model, we first present the general process of the localization method based on sparse representation. In the off-line fingerprint map construction stage, the RSS samples in the fingerprint maps are represented as a redundant dictionary. In the online localization stage, the RSS measurement is sparsely represented on the redundant dictionary, and its location is estimated by the positions of the RSS samples with non-zero sparse coefficients. The following gives the details of the localization procedure.

### 3.1. Off-Line Fingerprint Maps Construction Stage

In the off-line fingerprint map construction stage, assuming that there are *M* wireless Access Points (APs) and *N* positions with known coordinates in the scene, the RSS signal strengths of these APs are collected, so we could form an M×N fingerprint maps matrix as below:

(1) $$\begin{array}{c} \Phi =\left(\begin{array}{cccc} \phi_{1,1} & \phi_{1,2} & \cdots & \phi_{1,N}\\ \phi_{2,1} & \phi_{2,2} & \cdots & \phi_{2,N}\\ \cdots & \cdots & \cdots & \cdots \\ \phi_{M,1} & \phi_{M,2} & \cdots & \phi_{M,N} \end{array}\right) \end{array}$$

where $\phi_{i,j}$ represents the signal strength of the *i*-th AP on the *j*-th position. Each column vector $\phi_{j}\in {ℜ}^{M}$ represents the signal strengths of the *M* APs on the *j*-th position. The signal strength $\phi_{j}$ and its position $\left(u_{j},v_{j}\right)$ are recorded as the fingerprint maps of the scene, denoted by $\left\{\left(u_{j},v_{j};\phi_{j}\right)|j=1,\ldots ,N\right\}$. In practice, if the RSS signal strength of an AP is not received at a certain position, the RSS signal strength is set to −100 dBm to guarantee the completeness of the fingerprint maps matrix.

### 3.2. Online Localization Stage

To obtain the locations of the set of RSS measurements $Y={\left[y_{1},y_{2},\ldots ,y_{K}\right]}^{T}$ on the walking path, it was first matched with the fingerprint maps matrix **Φ** by the following sparse representation model:

(2) $$\begin{array}{c} \min_{X}\;{∥X∥}_{0},\;s.t.\;Y=\Phi X, \end{array}$$

where ${∥\cdot ∥}_{0}$ represents the $\ell_{0}$ norm, X is the sparse representation of Y with the element $x_{ij}$ representing the similarity between the measurement $y_{j}$ and the RSS sample $\phi_{i}$ in the fingerprint maps. In general, the non-convex $\ell_{0}$ norm induces some optimization difficulty; hence, one usually uses the surrogate $\ell_{1}$ norm instead. Therefore, a new localization model with $\ell_{1}$ sparse norm is formulated as the following equation:

(3) $$\begin{array}{c} \min_{X}\;{∥X∥}_{1},\;s.t.\;Y=\Phi X, \end{array}$$

where ${∥X∥}_{1}=\sum_{i,j}\left|x_{ij}\right|$ is the $\ell_{1}$ norm of the coefficient matrix X. Furthermore, to yield a coordinate independent notion of sparsity, the following non-negative affine constraint is added to the usual sparse model.

(4) $$\begin{array}{c} y_{j}=x_{1j}\phi_{1}+x_{2j}\phi_{2}+\ldots +x_{Nj}\phi_{N},\;x_{1j}+x_{2j}+\ldots +x_{Nj}=1,\;x_{ij}\geq 0. \end{array}$$

Therefore, we could obtain a sparse representation model with a non-negative affine constraint as below:

(5) $$\begin{array}{c} \min_{X}\;{∥X∥}_{1},\;s.t.\;Y=\Phi X,\;\sum_{i}x_{ij}=1,\;x_{ij}\geq 0. \end{array}$$

Based on the above sparse representation, there is a basic framework of localization using the sparse coefficients, in which the location of an RSS measurement $y_{j}$ can be estimated from its sparse coefficient $x_{j}={\left[x_{1j},x_{2j},\ldots ,x_{Nj}\right]}^{T}$ as below:

(6) $$\begin{array}{c} \left[\begin{array}{c} u\\ v \end{array}\right]=\frac{\sum_{i}\max\left\{x_{ij}-r,0\right\}\left[\begin{array}{c} u_{i}\\ v_{i} \end{array}\right]}{\sum_{i}\max\left\{x_{ij}-r,0\right\}} \end{array}$$

where r>0 denotes the threshold of non-zero sparse representation coefficient for choosing the useful coefficients that are greater than *r*. This method is called the Basic Sparse Representation (B-SR) localization method. In the B-SR method, besides the non-negative affine constraint, there is no other constraint added to the sparse coefficient matrix X, which means that the intrinsic spatial and temporal correlations among the RSS samples and measurements are not investigated.

## 4. Localization Method Based on Sparse Representation with the Spatio-Temporal Constraint

To utilize the intrinsic spatial and temporal correlations among the RSS samples and measurements and obtain better localization result, we propose a Spatio-Temporal-constrained Sparse Representation (ST-SR) model for localization. In fact, the RSS measurements $Y=[y_{1},y_{2},\ldots ,y_{K}]$ are sampled in continuous time series, so the adjacent columns of Y should be correlative in the time domain and generally behave consistently and smoothly. It is natural to require the coefficient matrix X to preserve this property. Therefore, we introduce a temporal continuous constraint into the B-SR model and build a Temporal-constrained SR model (T-SR) for maintaining the consistency and smoothness among the adjacent columns. The T-SR model is formulated as follows,

(7) $$\begin{array}{c} \min_{X}{\;∥X∥}_{1}+\lambda_{1}{∥\text{XD}∥}_{F}^{2},\;s.t.\;Y=\Phi X,\;\sum_{i}x_{ij}=1,\;x_{ij}\geq 0, \end{array}$$

where $\lambda_{1}$ is a tunable parameter, ${∥\cdot ∥}_{F}$ denotes the matrix *Frobenius* norm, D is a K×(K−1) matrix, which is given as below:

(8) $$\begin{array}{c} D={\left(\begin{array}{ccccc} -1 & 0 & 0 & \cdots & 0\\ 1 & -1 & 0 & \cdots & 0\\ 0 & 1 & -1 & \cdots & 0\\ \vdots & \vdots & \ddots & \ddots & \vdots \\ 0 & 0 & 0 & \ddots & -1\\ 0 & 0 & 0 & \cdots & 1 \end{array}\right)}_{K\times (K-1),} \end{array}$$

and the item ${∥\text{XD}∥}_{F}^{2}$ enforces the consistency of the columns of X.

Besides the temporal correlation among the RSS measurement Y, which relates the temporal constraint to its sparse representation X, the spatial correlation among RSS samples also exists in the fingerprint maps **Φ**. That is, the RSS sample measured at a position has little difference from the ones measured in its local neighborhood. Mathematically, the RSS sample can be nearly represented by the linear combination of the other samples in its neighborhood. From this observation, for an RSS sample $\phi_{p}$ at the location $\left(u_{p},v_{p}\right)$, we define a neighborhood $O(u_{p},v_{p})$ of $\left(u_{p},v_{p}\right)$ as $\left\{\left(u_{q},v_{q}\right)|d\left(q,p\right)\leq r,q\neq p\right\}$, where $d\left(q,p\right)={\left({\left(u_{q}-u_{p}\right)}^{2}+{\left(v_{q}-v_{p}\right)}^{2}\right)}^{\frac{1}{2}}$ represents the distance between $\left(u_{p},v_{p}\right)$ and $\left(u_{q},v_{q}\right)$, and *r* is the default neighborhood size. Therefore, the spatial correlation of RSS samples in the local neighborhood $O(u_{p},v_{p})$ can be expressed as below:

(9) $$\begin{array}{c} \phi_{p}\approx \sum_{q}s_{qp}\phi_{q},\;\forall \left(u_{q},v_{q}\right)\in O\left(u_{p},v_{p}\right), \end{array}$$

where $s_{qp}$ is the linear combination coefficient. For an RSS measurement $y_{j}$, it can be sparsely represented over the RSS samples in the the fingerprint maps, i.e., $y_{j}=x_{1j}\phi_{1}+x_{2j}\phi_{2}+\ldots +x_{Nj}\phi_{N}$, where the coefficient $x_{pj}$ can be explained as the similarity between $y_{j}$ and $\phi_{p}$. Therefore, if $\phi_{p}$ and $\phi_{q}$ have spatial correlation in Equation (9), then the corresponding coefficients $x_{pj}$ and $x_{qj}$ should also have a similar correlation, i.e., the rows $X_{p}={\left\{x_{p1},\ldots ,x_{pM}\right\}}^{T}$ and $X_{q}={\left\{x_{q1},\ldots ,x_{qM}\right\}}^{T}$ of X have the following equation:

(10) $$\begin{array}{c} X_{p}\approx \sum_{q}s_{qp}X_{q},\;\forall \left(u_{q},v_{q}\right)\in O\left(u_{p},v_{p}\right). \end{array}$$

Therefore, we could introduce the above spatial constraint into the B-SR model and build a spatial constrained SR model (S-SR) as follows:

(11) $$\begin{array}{c} \min_{X}{\;∥X∥}_{1}+\lambda_{2}{∥\text{SX}∥}_{F}^{2},\;s.t.\;Y=\Phi X,\;\sum_{i}x_{ij}=1,\;x_{ij}\geq 0, \end{array}$$

where $\lambda_{2}$ is a tunable parameter and S is the matrix composed of elements of $s_{qp},q=1,\ldots ,N,$p=1,…,N, which is used for maintaining the spatial correlation among the RSS samples in the fingerprint maps. To this end, the key problem is how to design a proper S to describe the spatial correlation. Generally, the spatial correlation of the RSS samples in the fingerprint maps depends on their positions and signal strength values. Therefore, we simply define S according to the similarity of the signal strength and the above local neighborhood. That is,

(12) $$S_{qp}=\left\{\begin{array}{cc} 1, & \text{if}\;q=p,\\ -\frac{∥\phi_{q}-\phi_{p}{∥}_{2}}{\sum_{\left(u_{i},v_{i}\right)\in O\left(u_{p},v_{p}\right)}{∥\phi_{i}-\phi_{p}∥}_{2}}, & \text{if}\;q\neq p\;and\;\left(u_{q},v_{q}\right)\in O\left(u_{p},v_{p}\right),\\ 0, & \text{otherwise}. \end{array}\right.$$

After determining the temporal and spatial constraints matrix D and S, we combine the T-SR model in Equation (7) and the S-SR model in Equation (11) together and form a novel Spatio-Temporal-constrained Sparse Representation (ST-SR) model for indoor localization as below:

(13) $$\begin{array}{c} \min_{X}{\;∥X∥}_{1}+\lambda_{1}{∥\text{XD}∥}_{F}^{2}+\lambda_{2}{∥\text{SX}∥}_{F}^{2},\;s.t.\;Y=\Phi X,\;\sum_{i}x_{ij}=1,\;x_{ij}\geq 0. \end{array}$$

Furthermore, we could relax the signal representation condition Y=ΦX in the model and transform it into the objective function as a reconstruction error item. Therefore, we get the final ST-SR model as below:

(14) $$\begin{array}{c} \min_{X}{\;∥X∥}_{1}+\lambda_{1}{∥\text{XD}∥}_{F}^{2}+\lambda_{2}{∥\text{SX}∥}_{F}^{2}+\lambda_{3}{∥Y-\Phi X∥}_{F}^{2},\;s.t.\;\sum_{i}x_{ij}=1,\;x_{ij}\geq 0, \end{array}$$

where $\lambda_{3}$ is a tunable parameter. In the next section, we will give the solution to this model.

## 5. Optimization Solution to the ST-SR Model

The model in Equation (14) is a complex optimization problem and is difficult to solve directly. For this reason, we adopt the alternating direction method of multipliers (ADMM) [33] to solve it. Firstly, we introduce two extra variables A and Z and let A=X, Z=X. Therefore, the problem in Equation (14) can be reformulated as the following problem with the introduced linear constraints:

(15) $$\begin{array}{c} \min_{Z,A,X}{\;∥Z∥}_{1}+\lambda_{1}{∥\text{AD}∥}_{F}^{2}+\lambda_{2}{∥\text{SX}∥}_{F}^{2}+\lambda_{3}{∥Y-\Phi X∥}_{F}^{2},\;s.t.\;\sum_{i}x_{ij}=1,\;z_{ij}\geq 0,\;Z=X,A=X. \end{array}$$

Then, we construct the following objective function of the above problem by the augmented Lagrangian multiplier method.

(16) $$\begin{array}{cc} L(Z,A,X,F_{1},F_{2},F_{3},\gamma )= & {∥Z∥}_{1}+\lambda_{1}{∥\text{AD}∥}_{F}^{2}+\lambda_{2}{∥\text{SX}∥}_{F}^{2}+\lambda_{3}{∥Y-\Phi X∥}_{F}^{2}\\ & +\langle F_{1},Z-X\rangle +\langle F_{2},A-X\rangle +\langle F_{3},\text{bX}-c\rangle \\ & +\frac{\gamma }{2}{(∥Z-X∥}_{F}^{2}{+∥A-X∥}_{F}^{2}{+∥\text{bX}-c∥}_{F}^{2}), \end{array}$$

where $F_{1}$, $F_{2}$ and $F_{3}$ are the Lagrangian multipliers, γ>0 is an adaptive weight parameter, $b=1^{1\times N}$, $c=1^{1\times K}$ and 1 denotes the row vector with all elements being one. Here Z is confined by the non-negative constraint $z_{ij}\geq 0$, denoted by Z≥0. For convenience, we rewrite this function into the following form:

(17) $$\begin{array}{cc} L(Z,X,A,F_{1},F_{2},F_{3},\gamma )= & {∥Z∥}_{1}-\frac{1}{\gamma }(∥F_{1}{∥}_{F}^{2}+∥F_{2}{∥}_{F}^{2}+{∥F∥}_{3}^{2})+h\left(Z,X,A,F_{1},F_{2},F_{3},\gamma \right), \end{array}$$

where $h\left(Z,X,A,F_{1},F_{2},F_{3},\gamma \right)=\lambda_{1}{∥\text{AD}∥}_{F}^{2}+\lambda_{2}{∥\text{SX}∥}_{F}^{2}+\lambda_{3}{∥Y-\Phi X∥}_{F}^{2}+\frac{\gamma }{2}(∥Z-X+\frac{F_{1}}{\gamma }{∥}_{F}^{2}+∥A-X+\frac{F_{2}}{\gamma }{∥}_{F}^{2}+∥\text{bX}-c+\frac{F_{3}}{\gamma }{∥}_{F}^{2})$. For the objective function in Equation (17), we adopt the linearized alternating direction method in [33] to solve by an iteration procedure. The following steps give the details of the iterations of Z,A,X and other parameters. We use *t* to denote the current iteration.

### 5.1. Update Z while Fixing A and X

When A and X are fixed, the objective function in Equation (17) is degenerated into a function with respect to Z. Therefore, we can solve Z by the following optimization problem:

(18) $$\begin{array}{cc} Z^{t+1} & =\arg\min_{Z\geq 0}{\;∥Z∥}_{1}+\frac{\gamma^{t}}{2}{∥Z-X^{t}+\frac{F_{1}^{t}}{\gamma^{t}}∥}_{F}^{2}\\ & =\arg\min_{Z\geq 0}\;\tau^{t}{∥Z∥}_{1}+\frac{1}{2}{∥Z-{\hat{Z}}^{t}∥}_{F}^{2}, \end{array}$$

where $\tau^{t}=\frac{1}{\gamma^{t}}$, ${\hat{Z}}^{t}=X^{t}-\frac{F_{1}^{t}}{\gamma^{t}}$. According to the conclusions in [34,35], the closed-form solution to the problem is given by the following form:

(19) $$\begin{array}{c} Z^{t+1}=\max\left\{{\hat{Z}}^{t}-\tau^{t},0\right\}. \end{array}$$

### 5.2. Update A while Fixing Z and X

When Z and X are fixed, the objective function in Equation (17) is degenerated into a function with respect to A. Therefore, we can solve A by the following optimization problem:

(20) $$\begin{array}{cc} A^{t+1} & =\arg\min_{A}\;h\left(Z^{t+1},X^{t},A,F_{1}^{t},F_{2}^{t},F_{3}^{t},\gamma^{t}\right). \end{array}$$

Let $\frac{\partial h}{\partial A}=0$. Then, we have the closed-form solution of A of the following form:

(21) $$\begin{array}{c} A^{t+1}=\gamma \left(X^{t}-\frac{F_{2}^{t}}{\gamma }\right){\left(2\lambda_{1}{\mathrm{DD}}^{T}+\gamma^{t}I_{1}\right)}^{-1}, \end{array}$$

where $I_{1}\in {ℜ}^{K\times K}$ is an identity matrix.

### 5.3. Update X while Fixing Z and A

When Z and A are fixed, the objective function in Equation (17) is degenerated into a function with respect to X. Therefore, we can solve X by the following optimization problem:

(22) $$\begin{array}{cc} X^{t+1} & =\arg\min_{X}\;h\left(Z^{t+1},X,A^{t+1},F_{1}^{t},F_{2}^{t},F_{3}^{t},\gamma^{t}\right). \end{array}$$

Let $\frac{\partial h}{\partial X}=0$. Then, we also have the closed-form solution of X of the following form:

(23) $$\begin{array}{c} X^{t+1}={\left(2\lambda_{2}S^{T}S+2\lambda_{3}\Phi^{T}\Phi +\gamma^{t}\left(2I_{2}+b^{T}b\right)\right)}^{-1}\left(2\lambda_{3}\Phi^{T}Y+\gamma^{t}\left(\left(Z^{t+1}+\frac{F_{1}^{t}}{\gamma^{t}}\right)+\left(A^{t+1}+\frac{F_{2}^{t}}{\gamma^{t}}\right)+b^{T}\left(c-\frac{F_{3}^{t}}{\gamma^{t}}\right)\right)\right), \end{array}$$

where $I_{2}\in {ℜ}^{N\times N}$ is also an identity matrix.

### 5.4. Update the Multiplier $F_{1}$, $F_{2}$, $F_{3}$ and Parameter *γ*

After updating the coefficient matrix A,X,Z at each iteration, the multipliers $F_{1}$, $F_{2}$, $F_{3}$ and parameter *γ* are updated by the following formulas:

(24) $$\begin{array}{c} F_{1}^{t+1}=F_{1}^{t}+\gamma^{t}\left(Z^{t+1}-X^{t+1}\right). \end{array}$$

(25) $$\begin{array}{c} F_{2}^{t+1}=F_{2}^{t}+\gamma^{t}\left(A^{t+1}-X^{t+1}\right). \end{array}$$

(26) $$\begin{array}{c} F_{3}^{t+1}=F_{3}^{t}+\gamma^{t}\left({\text{bX}}^{t+1}-c\right). \end{array}$$

(27) $$\begin{array}{c} \gamma^{t+1}=\min\left\{\rho \gamma^{t},\gamma^{\max}\right\}, \end{array}$$

where ρ=1.1, $\gamma^{\max}=10^{10}$. In our algorithm, the stopping criterion is measured by the following condition:

(28) $$\begin{array}{c} \max\left\{\begin{array}{c} ∥Z^{t+1}-Z^{t}{∥}_{\infty },∥A^{t+1}-A^{t}{∥}_{\infty },{∥X^{t+1}-X^{t}∥}_{\infty },\\ ∥Z^{t+1}-X^{t+1}{∥}_{\infty },∥A^{t+1}-X^{t+1}{∥}_{\infty },{∥{\text{bX}}^{t+1}-c∥}_{\infty } \end{array}\right\}\leq \varepsilon , \end{array}$$

where ${∥\cdot ∥}_{\infty }$ denotes the infinite norm.

Iteratively updating Z, A and X will form a solution to Equation (14). Integrating the above iterations, the solution to Equation (14) is obtained, and the complete algorithm is summarized in Algorithm 1. As suggested in [36], we can make Steps 1–5 in the algorithm parallel. Additionally, from the closed-form solutions to each subproblem for Z, A and X, the overall convergence of the algorithm can be guaranteed.
**Algorithm 1** Solving the proposed ST-SR model by ADMMInitialize:
$Z^{0}=X^{0}=A^{0}=0^{N\times K}$, $F_{1}^{0}=1^{N\times K}$, $F_{2}=1^{N\times K}$, $F_{3}^{0}=1^{1\times N}$, $\gamma^{0}=10^{-2}$, ρ=1.1, $\gamma^{\max}=10^{10}$, $\varepsilon =10^{-6}$, the number of maximum iteration MaxIter=1000, set t=0.Input: The fingerprint maps matrix **Φ**, the temporal constraint matrix D, the spatial constraint matrix S, the tunable parameter $\lambda_{1}$, $\lambda_{2}$ and $\lambda_{3}$;**while** not converged and t≤MaxIter
**do**
1)Calculate $Z^{t+1}$ by Equations (18) and (19);2)Calculate $A^{t+1}$ by Equations (20) and (21);3)Calculate $X^{t+1}$ by Equations (22) and (23);4)Calculate $F_{1}^{t+1}$, $F_{2}^{t+1}$ and $F_{3}^{t+1}$ by Equations (24)–(26);5)Calculate $\gamma^{t+1}$ by Equation (27);6)Check the convergence condition defined as Equation (28);7)t=t+1.**end while**Output: The matrices Z, A and X.


The computational cost of our proposed algorithm is mainly determined by [33]. In each iteration, the soft thresholding to update the sparse matrix $Z\in {ℜ}^{N\times K}$ has a complexity of O(NK). The complexity of updating matrix $A\in {ℜ}^{N\times K}$ and $X\in {ℜ}^{N\times K}$ is $O(K^{2}N)$ and $O(N^{2}K)$, respectively. Therefore, the cost of all iterations is $O(t\left(NK+K^{2}N+N^{2}K\right))$.

## 6. Experiments

To evaluate the proposed ST-SR model-based localization method, several experiments are implemented in both the simulated scene and a real environment. The experimental results are compared with state-of-the-art localization methods, including the classic KNN method, the Compressive Sensing (CS)-based method [15], the Basic Sparse Representation (B-SR)-based method [10], and the methods of Temporal-Sparse Representation (T-SR) and Spatial-Sparse Representation (S-SR), which only use the temporal or spatial constraint in the sparse representation model. In our experiments, each parameter $\lambda_{i}$ is related to the experiment scenarios. By a set of experiments with different parameter settings (for each $\lambda_{i}$, 30 values are tested), we select the optimal parameters according to the experimental results.

### 6.1. Experiments in the Simulated Scene

The simulated scene is a 30 m × 30 m square. We first randomly deployed 15 APs (M=15) in the scene. The RSS distribution of each AP is determined by the following wireless signal attenuation model [37]:

(29) $$P_{r}\left(d\right)=P_{t}\left(d\right)-P\left(d_{0}\right)-10n\log_{10}{\left(\frac{d}{d_{0}}\right)}^{n}-U_{\sigma }$$

where $P_{r}\left(d\right)$ indicates the signal intensity received at distance *d* from the AP, $P_{t}\left(d\right)$ is the transmitting intensity at distance *d* from the AP, $P(d_{0})$ is the average signal strength loss value at the reference distance $d_{0}$, which is usually set as 1 m, *n* is the given path-loss exponent and $U_{\sigma }$ denotes the Gauss noise with distribution N(0,σ). When we get the distance between the sampling position and the AP and the path loss exponent, the RSS value at a position could be calculated. We assume that the maximal signal transmission distance is 30 m, which means the RSS value would be set to the minimal value of −100 dB if the distance is larger than 30 m. In our experiment, we set the path loss exponent n=4.4, the average signal strength loss value $\bar{P}\left(d_{0}\right)=-35$ dB and the variance of the Gauss noise σ=30.

**Figure 1:**
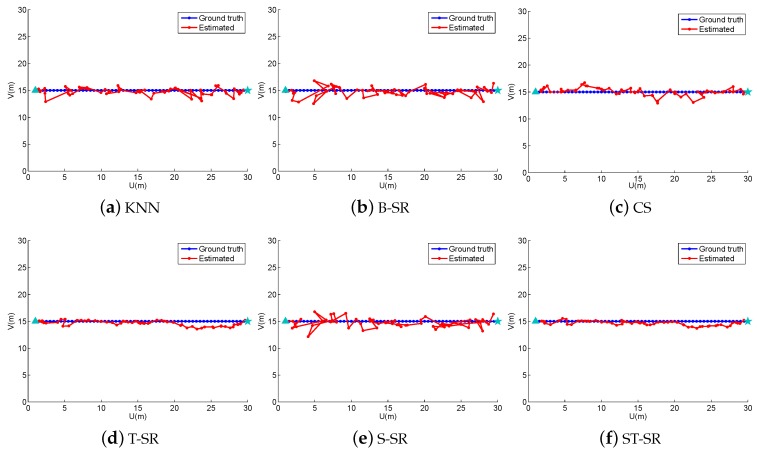
The localization results (red) of the line path (blue) in the simulated scene. B-SR, Basic Sparse Representation; CS, Compressed Sensing; ST-SR, Spatio-Temporal Sparse Representation.

**Figure 2:**
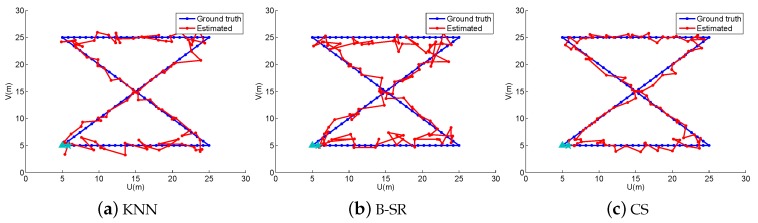

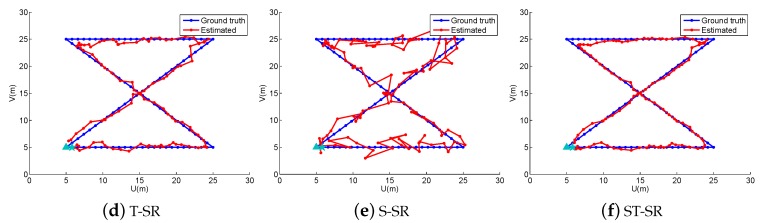
The localization results (red) of the “8” path (blue) in the simulated scene.

**Figure 3:**
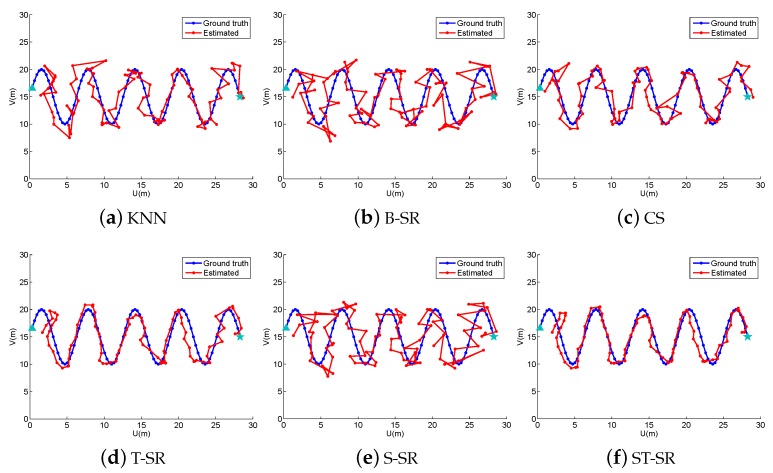
The localization results (red) of the snake path (blue) in the simulated scene.

**Figure 4:**
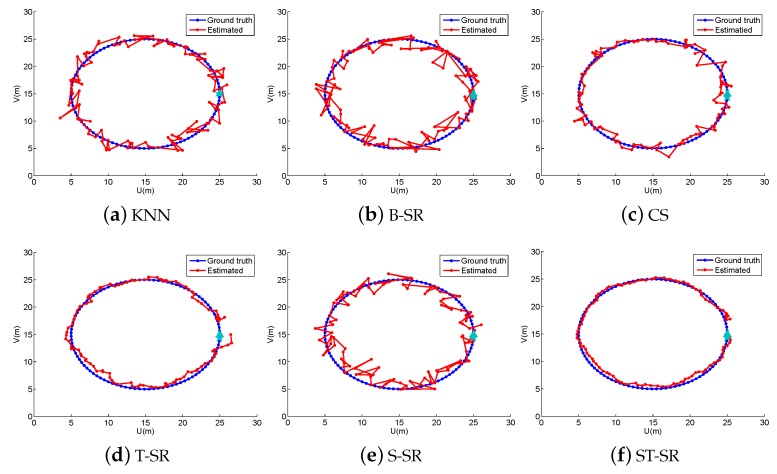
The localization results (red) of the circle path (blue) in the simulated scene.

In the off-line fingerprint maps construction stage, we collect 900 RSS samples (N=900) as the fingerprint maps at the cross positions in a uniform grid of 30×30 with a space of 1 m both in the horizontal and vertical directions in the simulated area. Thus, we construct a fingerprint maps matrix $\Phi \in {ℜ}^{15\times 900}$. In the online localization stage, we design four walking paths, the line path, the “8” path, the snake path and the circle path (shown in Figure 1, Figure 2, Figure 3 and Figure 4, respectively). The beginning point is located at the position of “△”, and the end point is denoted by “★”. From the beginning point to the end point, we set the measurement points (denoted by “·”) on the path with a stepsize of 1 m to get the observing RSS measurements. To evaluate the accuracy of the localization results, we define the localization error as the average error between the estimation location and the ground truth at all measurement points:

(30) $$err=\frac{\sum_{i}{\left({\left(u_{i}^{*}-u_{i}\right)}^{2}+\left(v_{i}^{*}-v_{i}\right)\right)}^{\frac{1}{2}}}{K}$$

where $\left(u_{i}^{*},v_{i}^{*}\right)$ and $\left(u_{i},v_{i}\right)$ represent the estimated coordinate and the real coordinate of the *i*-th measurement point, *K* is the total number of the measurement points on the path.

**Figure 5:**
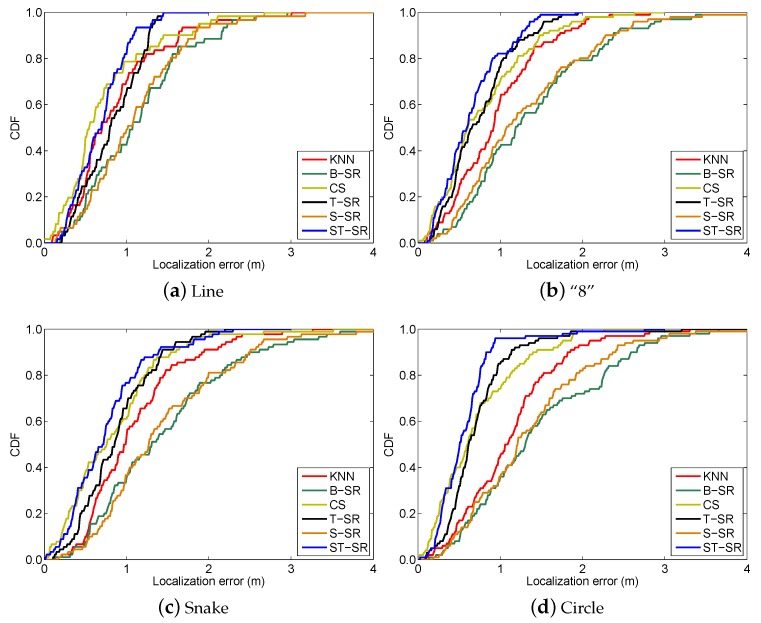
The CDF curves of the localization error for all the methods in the simulated scene.

To obtain reliable results, each experiment has been repeated 20 times, and the average result is recorded as the final result. The parameters of the proposed ST-SR, S-SR and T-SR methods are empirically set as $\lambda_{1}=200$, $\lambda_{2}=2$, $\lambda_{3}=1$. Figure 1, Figure 2, Figure 3 and Figure 4 show the estimated paths (the red line) compared with the ground truth (the blue line) with different localization methods. Intuitively, the proposed ST-SR method has the best localization performance, as it has a close and smooth estimated path compared with the ground truth. The localization errors of different methods are reported in Table 1. Meanwhile, we show the empirical Cumulative Distribution Function (CDF) curves of the localization error for all of the methods in Figure 5. It is shown that the proposed ST-SR method obtains the best location results (in bold text) and shows more robustness than the other methods.

### 6.2. Experiment in a Real Scene

**Figure 6:**
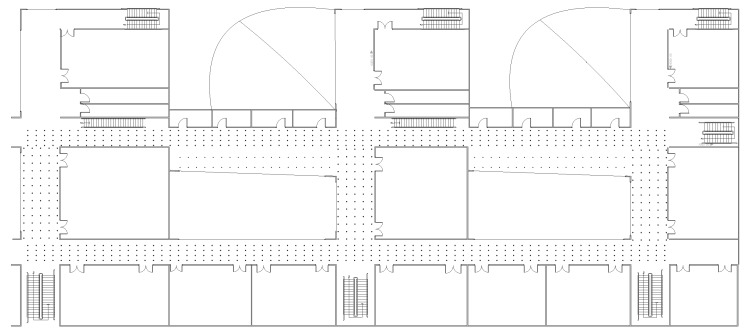
The real scene for localization and the positions (the black dots) at which the RSS signal strengths are recorded to construct the RSS fingerprint maps.

To evaluate our proposed method in a real scene, we choose the third layer of a teaching building in our university as the experimental area for localization. The size of the experimental area is 67 × 15 m, as shown in Figure 6.

Similar to the experiment in the simulated scene, we first construct the RSS fingerprint maps. For this purpose, an experimenter carries a mobile terminal to record the RSS signal strength of the APs in the area. We have detected 30 APs in this area, so we set the number of APs M=30. We select 573 positions in total in this area to measure the RSS signal strength. Shown as the black dots in Figure 6, these positions usually are located uniformly in the area with a spacing of about 1.2 m. To avoid measurement error and obtain the accurate RSS value, the RSS signal strength is measured 10 times at each position, and the average value is recorded as the final RSS value. Finally, we construct a fingerprint map matrix from the selected 573 positions, denoted by $\Phi \in {ℜ}^{30\times 573}$.

**Figure 7:**
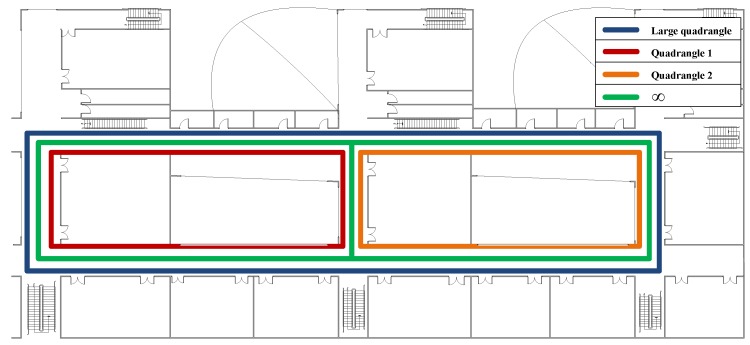
The walking paths in the real scene.

**Figure 8:**
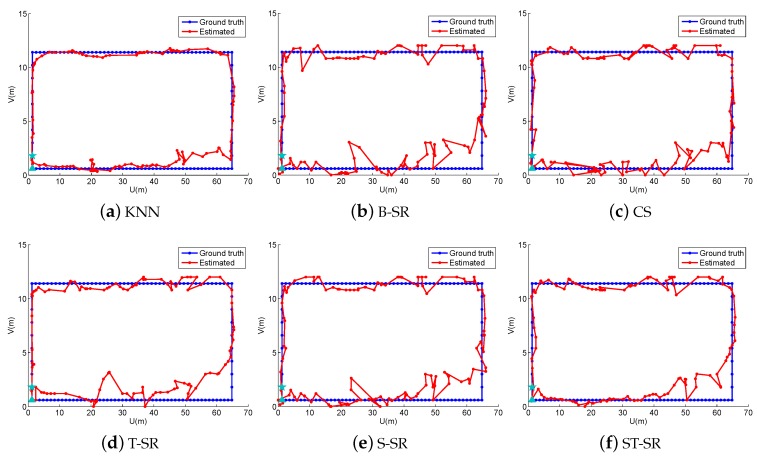
The localization results (red) of the large quadrangle path (blue) in the real scene.

**Figure 9:**
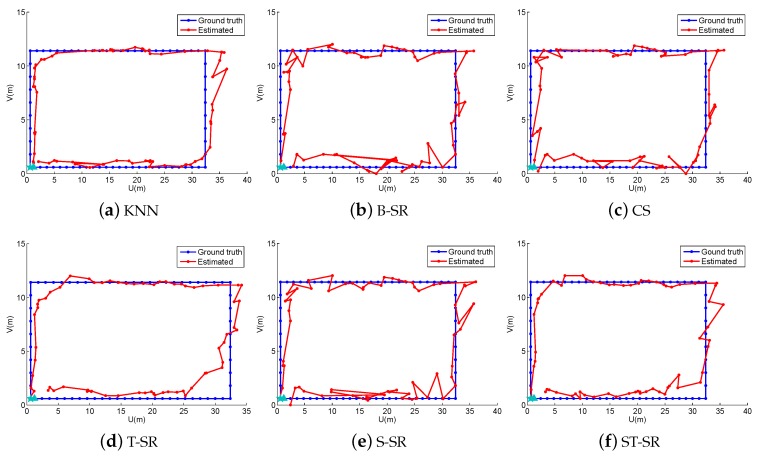
The localization results (red) of Quadrangle 1 path (blue) in the real scene.

**Figure 10:**
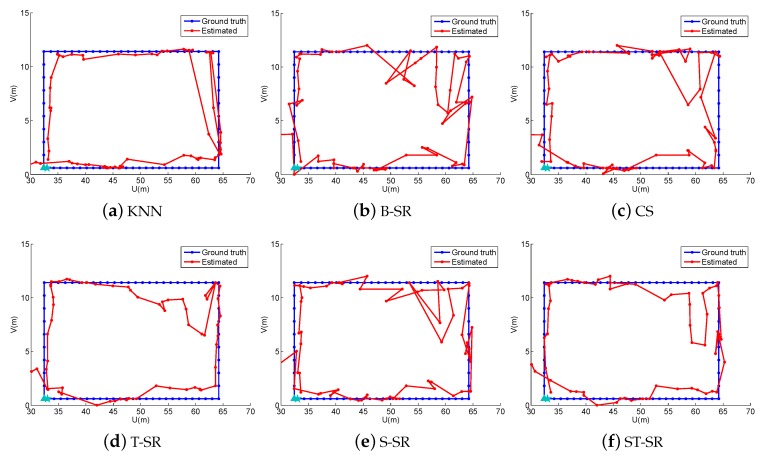
The localization results (red) of Quadrangle 2 path (blue) in the real scene.

**Figure 11:**
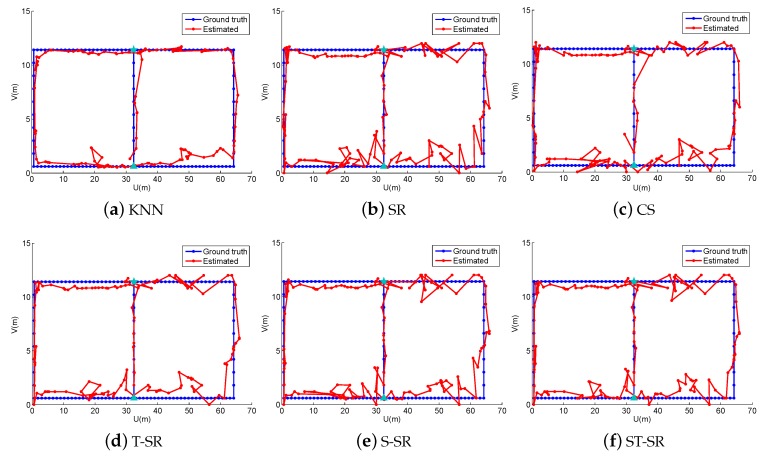
The localization results (red) of the “∞” path (blue) in the real scene.

**Figure 12:**
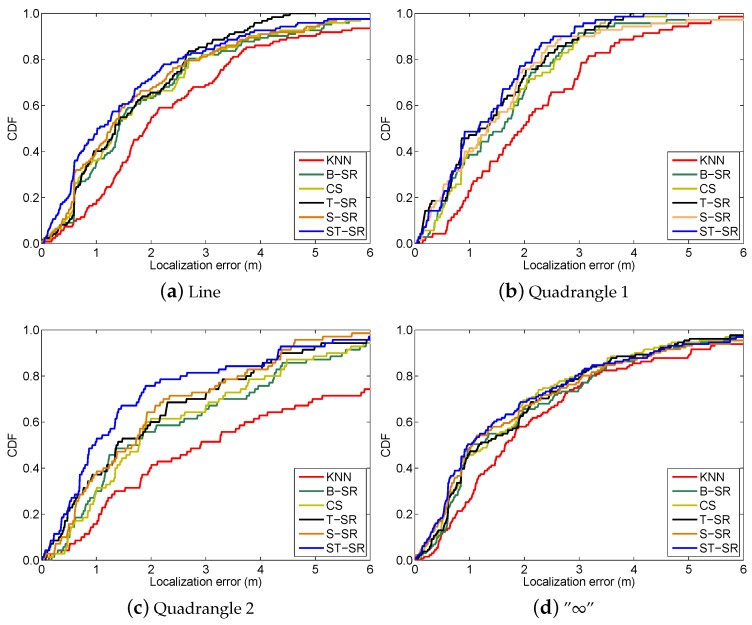
The CDF curves of the localization error for all of the methods in the real scene.

In the online localization stage, we also design four walking paths, the large quadrangle, Quadrangle 1, Quadrangle 2 and the “∞” path, shown as different color lines in Figure 7 and also shown in Figure 8, Figure 9, Figure 10 and Figure 11, respectively. We let an experimenter carry a mobile phone and walk in each path to record the RSS measurements at each measurement point. In our experiments, the distance between two adjacent measurement points is set to 1.2 m. Here, the experimental parameters $\lambda_{1}=180$, $\lambda_{2}=3$, $\lambda_{3}=1$. Each experiment is also repeated 20 times, and the average value is recorded as the final result. Figure 8, Figure 9, Figure 10 and Figure 11 show the estimated paths (the red line) and the ground truth (the blue line) with different localization methods. The localization errors of different methods are shown in Table 2. It is shown that the localization performance of all methods decreases in the real scene. It could be explained that the RSS values in the simulated scene are ideal without environment interference, but the RSS measurements in the real scene generally have much more interference factors, such as signal interference and diffraction. Even so, the proposed ST-SR method obtains the best location results (in bold text) and performs much more robustly than others, as shown in Table 2. Similar to the experiments in the simulation scene, in Figure 12, we show the CDF curves of the localization error for all of the methods in the real scene.

From the above experimental results in both the simulated and real scenes, it is shown that our proposed ST-SR method has better localization performance with more robustness compared with other localization methods. The improvement is considered to benefit from the proposed temporal and spatial constraints introduced into the classic sparse method. These constraints make a proper formulation to describe the intrinsic spatial correlation of the RSS samples in the localization area and the temporal continuity of the RSS measurements when the user is moving.

## 7. Conclusions

In this paper, we proposed an indoor localization method based on sparse representation with a spatio-temporal constraint. Different from the basic sparse representation-based localization method, the temporal continuity of measurements from the moving user and the spatial correlation among the RSS samples of the fingerprint maps are introduced into the conventional B-SR model. The experimental results indicate that the proposed method has better localization results compared with the relevant methods.

In our experiments, both the simulated and real scenes assume that the localization area is a plane, without considering the more complex 3D scene. In future work, to obtain a more practical localization method, we will consider the localization problem in the real complicated environment, such as a multi-floor building with a complex inner structure. Meanwhile, there is no delicate preprocessing approach used in cleaning the RSS measurements data, which would decrease the localization performance caused by environment interference or noise. Therefore, another possible future work is to develop more effective data preprocessing methods to further improve the localization performance.

## Figures and Tables

**Table 1:** The localization errors (m) in the simulated scene.

Methods	KNN	B-SR	CS	T-SR	S-SR	ST-SR
Line	0.861 ± 0.572	1.124 ± 0.631	0.733 ± 0.563	0.814 ± 0.356	1.099 ± 0.685	**0.693 ± 0.395**
“8”	0.947 ± 0.546	1.374 ± 0.851	0.782 ± 0.550	0.744 ± 0.413	1.313 ± 0.897	**0.721 ± 0.409**
Snake	1.087 ± 0.598	1.452 ± 0.850	0.831 ± 0.599	0.877 ± 0.446	1.384 ± 0.849	**0.796 ± 0.389**
Circle	1.115 ± 0.600	1.448 ± 0.823	0.703 ± 0.512	0.710 ± 0.430	1.425 ± 0.775	**0.630 ± 0.278**
Average	1.002 ± 0.573	1.349 ± 0.706	0.762 ± 0.556	0.786 ± 0.411	1.372 ± 0.776	**0.710 ± 0.368**

**Table 2:** The localization errors (m) in the real scene.

Methods	KNN	B-SR	CS	T-SR	S-SR	ST-SR
Large quadrangle	2.420 ± 1.764	2.090 ± 1.905	1.881 ± 1.648	1.648 ± 1.137	1.942 ± 1.865	**1.560 ± 1.022**
Quadrangle 1	2.198 ± 1.402	1.882 ± 2.268	1.574 ± 1.052	1.426 ± 1.049	1.826 ± 2.097	**1.245 ± 0.710**
Quadrangle 2	3.834 ± 3.076	2.461 ± 2.044	2.392 ± 1.861	2.044 ± 1.719	2.066 ± 1.685	**1.845 ± 1.366**
“∞”	2.328 ± 2.161	1.975 ± 1.898	1.822 ± 1.774	1.830 ± 1.575	1.944 ± 1.752	**1.725 ± 1.513**
Average	2.695 ± 2.101	2.102 ± 2.029	1.917 ± 1.584	1.737 ± 1.370	1.695 ± 1.850	**1.595 ± 1.153**

## Abbreviations

The following abbreviations are used in this manuscript:

RSS	Received Signal Strength
B-SR	Basic Sparse Representation
T-SR	Temporal Sparse Representation
S-SR	Spatial Sparse Representation
ST-SR	Spatio-Temporal Sparse Representation
CS	Compressive Sensing
